# Identification of Androgen Receptor as a Molecular Docking Target for Survival and Response to Metformin‐Induced Ferroptosis in Liver Cancer

**DOI:** 10.1002/cnr2.70245

**Published:** 2025-06-30

**Authors:** Bin Zhang, Zehao Yu, Jinghui Zhang, Yini Xu, Mengna Zhang, Zhiqi Dai, Jiyun Zhu, Siming Zheng

**Affiliations:** ^1^ Hepatopancreatobiliary Surgery Department The First Affiliated Hospital of Ningbo University Ningbo China; ^2^ Health Science Center, Ningbo University Ningbo China; ^3^ Zhejiang University School of Medicine, Zhejiang University Hangzhou China

**Keywords:** ferroptosis, HCC, metformin, pharmacochemistry network

## Abstract

**Background:**

Hepatocellular Carcinoma (HCC) ranks among the most prevalent human cancers and stands as the third most common cause of death related to cancer globally. Current therapies for HCC include surgical resection, local ablation, chemoembolization, liver transplantation, and molecular‐targeted therapy. Only a small number of patients are detected in the early stage, and most patients are diagnosed at the time of the middle and late stages, thus losing the opportunity for surgical treatment, which is an essential reason for the high mortality of HCC patients. Initiating cytotoxicity in cancer cells stands as a fundamental approach for tumor treatment, with the majority of research centering on apoptosis.

**Aims:**

Since anti‐apoptotic methods often fulfill cancer cells' ability to resist anticancer drugs, research on new induction forms of regulative cell death, such as ferroptosis, is of great clinical value.

**Methods and Results:**

In this study, we employed a combination of in silico molecular docking and in vitro cell validation experiments to identify three ferroptosis suppressor genes, AR, HIF1A, and CA9, as promising components of a survival prognosis model during the metformin‐induced ferroptosis process in liver cancer. Further, we discovered that AR could achieve efficient molecular docking with Metformin among these genes. Additionally, cell experiments revealed that Metformin could downregulate the protein expression level of AR.

**Conclusion:**

This research has developed a prognostic model for ferroptosis suppressor genes through the analysis of the ferroptosis process induced by metformin in liver cancer. It also screened and validated AR as a potential molecular docking target for metformin.

## 1 Introduction

Hepatocellular carcinoma (HCC), a prevalent malignancy globally, exhibits significant morbidity and mortality rates. According to the World Health Organization, HCC accounts for over 841,000 new cases and 782,000 deaths annually worldwide, making it the fourth leading cause of cancer‐related mortality [1]. Notably, China bears the highest burden, contributing 55% of global HCC cases, with age‐standardized incidence and mortality rates reaching 17.7 and 16.4 per 100,000 population, respectively [2]. This disparity is attributed to high rates of hepatitis B/C infections, aflatoxin exposure, and metabolic disorders such as obesity and diabetes. Current treatment strategies for primary hepatic carcinoma (PHC) are categorized into surgical and non‐surgical approaches. Surgical resection and liver transplantation remain the gold standard for early‐stage HCC, offering 5‐year survival rates of 60%–70% in select patients. However, 80% of HCC patients present with advanced disease or significant liver stiffness due to underlying cirrhosis, limiting surgical options. Non‐surgical treatments include interventional therapy, chemoradiotherapy, immunotherapy, traditional Chinese medicine therapy, and molecular‐targeted therapy [3]. In China, 90% of HCC patients experience varying degrees of hepatitis and cirrhosis, necessitating comprehensive and individualized treatment strategies.

With advancements in medical technology and drug development, HCC therapy has evolved into combination treatments involving multiple drugs [4]. First‐line agents like Sorafenib and Lenvatinib inhibit tyrosine kinases (VEGFR, PDGFR, FGFR) to block tumor angiogenesis and proliferation [5]. Second‐line drugs (e.g., Regorafenib, Cabozantinib) target additional pathways such as MET and AXL, improving median survival by 2–3 months [6]. However, drug resistance frequently arises through activation of the PI3K/AKT/mTOR pathway or epithelial‐mesenchymal transition (EMT) [7]. At the same time, the Atezolizumab (anti‐PD‐L1) + Bevacizumab (anti‐VEGF) combination has become the first‐line regimen for advanced HCC, enhancing tumor immunogenicity by suppressing Treg cells and normalizing tumor vasculature [8]. Nevertheless, only 30% of patients respond due to immunosuppressive microenvironments driven by TGF‐β and hypoxia‐inducible factors [9]. What is more, TACE combines ischemic injury with localized chemotherapy (e.g., Doxorubicin), achieving a median survival of 20–25 months in intermediate‐stage HCC [10]. 90Y radioembolization delivers β‐radiation via microspheres, inducing DNA damage through free radical generation [11]. However, post‐embolization hypoxia often activates HIF‐1α/GLUT1 pathways, promoting metastasis [12].

Given the advantages of the above‐mentioned drugs and treatment strategies, their limitations also represent major challenges currently faced by patients with liver cancer. Sorafenib resistance is linked to GPX4 upregulation, which suppresses lipid peroxidation and ferroptosis [13]. ICIs cause immune‐related adverse events (e.g., hepatitis, colitis) in 40% of patients, while TKIs induce hypertension and hand‐foot syndrome [14]. Also, intra‐tumoral cancer stem cells (CSCs) evade therapy via ALDH1A1‐mediated detoxification and Wnt/β‐catenin activation [15].

Ferroptosis is a form of regulated cell death that is iron‐dependent and characterized by the accumulation of lipid peroxides, offering a potential strategy to overcome resistance to apoptosis [16]. Hepatocellular carcinoma (HCC) cells demonstrate a heightened susceptibility to ferroptosis—often referred to as “ferroptosis vulnerability”‐which is primarily attributed to two factors. First, iron overload in HCC cells is evidenced by the upregulation of transferrin receptor 1 (TFRC) and downregulation of ferritin, contributing to an increased labile iron pool [17]. Second, these cells display a strong dependency on glutathione peroxidase 4 (GPX4); knockdown of GPX4 results in a tenfold increase in 4‐hydroxynonenal (4‐HNE), a toxic lipid peroxidation byproduct, thereby significantly enhancing ferroptosis sensitivity [18]. In the context of HCC, ferroptosis holds significant therapeutic potential due to its ability to target cancer cells with altered metabolism and resistance to apoptosis. The high oxidative stress and iron load in HCC cells make them particularly susceptible to ferroptosis. By exploiting these vulnerabilities, ferroptosis‐inducing agents may overcome the limitations of conventional therapies, providing a more effective strategy against HCC.

**FIGURE 1:**
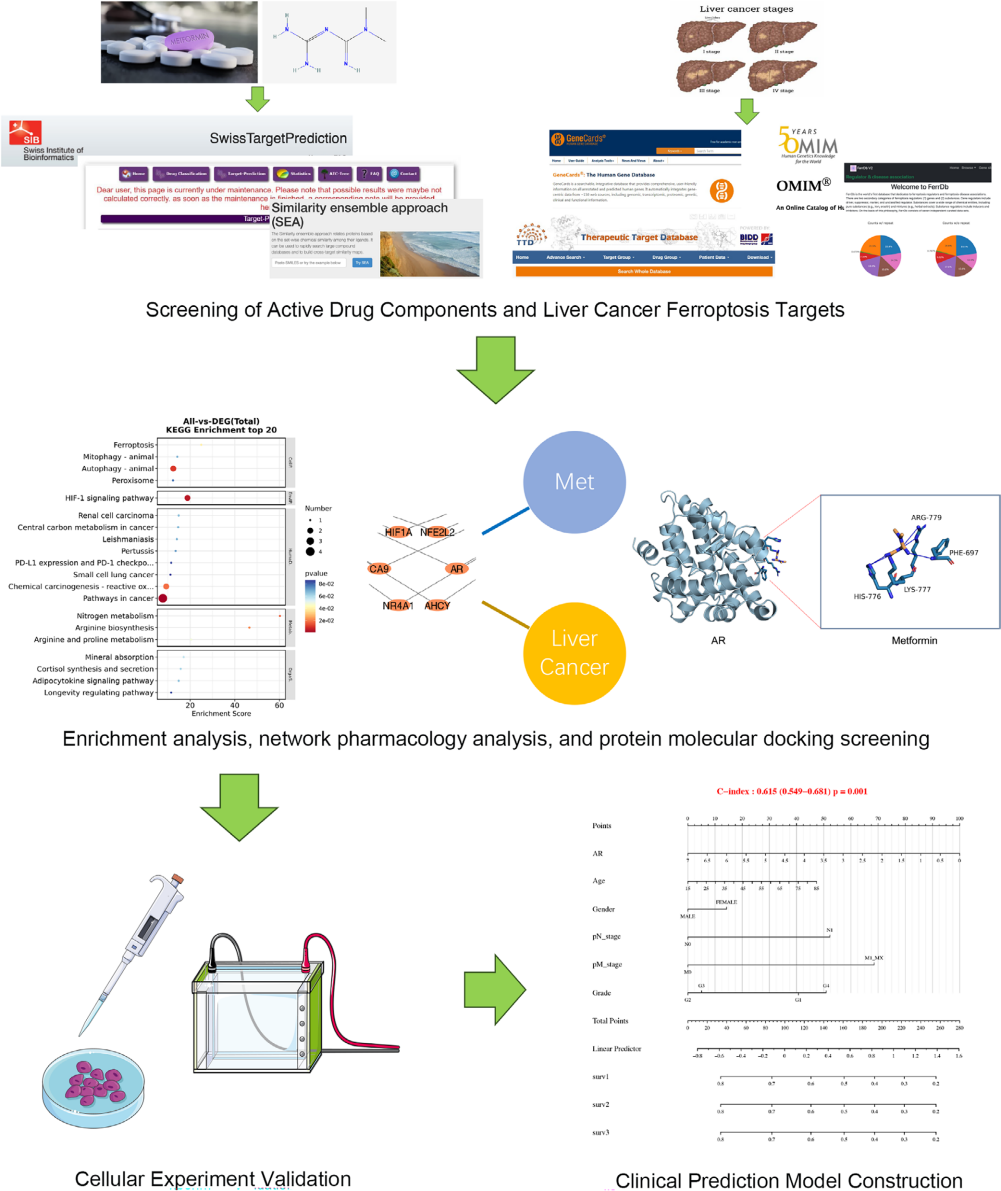
Workflow of the network pharmacological study of Metformin in treating Liver cancer.

This study utilized network pharmacology docking and in vitro cell experiment validation techniques to investigate and analyze the interaction between metformin and ferroptosis suppressor proteins in liver cancer. It identified the targets through which metformin promotes ferroptosis in liver cancer cells, providing new research directions for further in‐depth exploration of how metformin promotes ferroptosis in liver cancer and its long‐term clinical applications. Figure 1 summarized the methodologies of this study.

## 2 Materials and Methods

### 2.1 Forecasting the Potential Targets of the Constituents Identified in Metformin

We obtained all compound targets of Metformin from three compound–target databases (Swiss Target Prediction, SuperPred, Similarity Ensemble Approach). After filtering to remove duplicates, we obtained 151 drug targets. Lastly, gene symbols were standardized through the UniProt ID website (https://www.uniprot.org/) [19, 20, 21, 22].

### 2.2 Identification of Liver Cancer‐Related Ferroptosis Targets

We acquired liver cancer targets from the following databases: Online Mendelian Inheritance in Man (https://omim.org/), DisGeNet (https://www.disgenet.org/home/), Therapeutic Target Database (https://db.idrblab.net/ttd/), and GeneCards (https://www.genecards.org/home). The GeneCards database used a relevance score of ≥ 10 as a filtering criterion. After removing duplicates, we obtained 18 753 target genes for liver cancer. Additionally, we downloaded ferroptosis suppressor genes from the FerrDB V2 database, and after removing duplicates, we obtained 348 ferroptosis suppressor target genes. Then, through cross‐analysis, we identified 218 target genes for this study [23, 24, 25, 26, 27].

### 2.3 Building a Protein–Protein Interaction (PPI) Network

We utilized the OEBiotech Cloud Platform (https://cloud.oebiotech.com/#/bio/tools?cat_id&tag_id) to identify the intersecting target genes between the two datasets above. A protein–protein interaction (PPI) network was constructed on the STRING platform version 11.0 (https://string‐db.org) with a medium confidence threshold of 0.400 for subsequent research [28, 29].

### 2.4 Network of Drug, Compound, Target, and Disease (DCTD)

We imported the intersecting target genes, disease names, and Metformin into Cytoscape software version 3.9.0 to conduct a topological network analysis of the drug, active compounds, intersecting genes, and disease, thereby establishing a DCTD network model [30].

### 2.5 Analyses of Function and Pathway Enrichment

The enriched target genes identified in the results were subjected to Gene Ontology (GO), Kyoto Encyclopedia of Genes and Genomes (KEGG), and Reactome pathway enrichment analyses using R software and Bioconductor packages. The criteria for screening were established with a *p* value < 0.05 and a *q* value < 0.05 [31].

### 2.6 Molecular Docking of Compound–Target Interactions and Molecular Dynamics Analysis

The three‐dimensional (3D) configuration of Metformin was sourced from the PubChem database (https://pubchem.ncbi.nlm.nih.gov/). The conversion of format utilized OpenBabel, while molecular docking was executed with AutoDock Tools version 1.5.7. Visualization and analysis of the docking outcomes were carried out using the Protein‐Ligand Interaction Profiler. A docking score of less than −4.25 kcal/mol served as the threshold for molecular docking evaluation [32, 33].

Docking‐derived small molecule‐protein complexes were used as the initial structures for all‐atom MD simulations with AMBER 22. Small molecule charges were calculated using the Hartree–Fock (HF) SCF/6‐31G* method via the Antechamber module and Gaussian 09. GAFF2 and ff14SB force fields were applied to the small molecule and protein, respectively. Hydrogen atoms were added with the LEaP module, and the system was solvated in a truncated octahedral TIP3P water box (10 Å buffer) with Na+/Cl^−^ ions for charge neutrality. Energy minimization included 2500 steps each of steepest descent and conjugate gradient methods. The system was heated from 0 K to 298.15 K over 200 ps at constant volume, followed by 500 ps of NVT ensemble simulation to equilibrate solvent distribution and 500 ps of NPT equilibration at 1 atm and 298.15 K. A 100 ns NPT production simulation was conducted under periodic boundary conditions. Non‐bonded interactions were truncated at 10 Å, with electrostatics calculated using the Particle Mesh Ewald (PME) method. The SHAKE algorithm constrained hydrogen bond lengths, and the Langevin thermostat controlled temperature (collision frequency: 2 ps^−1^). Trajectories were saved every 10 ps for analysis.

The binding free energy between the protein and ligand was calculated using the MM/GBSA method based on 45–50 ns MD trajectories. The binding free energy was determined using the equation:

$${\Delta G}_{\text{bind}}={\mathrm{\Delta G}}_{\text{complex}}–\left({\mathrm{\Delta G}}_{\text{receptor}}+{\mathrm{\Delta G}}_{\text{ligand}}\right)$$

$$={\mathrm{\Delta E}}_{\text{internal}}+{\mathrm{\Delta E}}_{\mathrm{VDW}}+{\mathrm{\Delta E}}_{\text{elec}}+{\mathrm{\Delta G}}_{\mathrm{GB}}+{\mathrm{\Delta G}}_{\mathrm{SA}}$$

Entropy contributions were excluded due to computational cost and limited accuracy. This approach ensures efficient and reliable free energy calculations [34, 35, 36, 37, 38, 39, 40, 41, 42, 43, 44].

### 2.7 Cell Cultivation

In our laboratory, the human ovarian cell lines Huh7 and Hep3B were grown in DMEM supplemented with 10% FBS (Gibco, USA). These cell lines were acquired from Pricella and verified through DNA fingerprinting. Furthermore, all cell cultures were kept at 37°C with 5% CO2 in standard conditions and were passaged for less than two months following revival.

### 2.8 Antibodies and Drug

The following primary antibodies were used in this study: AR(AF6137) and GAPDH(AF7021) (Affinity, China). Metformin was obtained from the MCE (Shanghai, China).

### 2.9 Colony Formation Test

In a 6 well plate, cells were plated in three replicates at a density of 200 cells per well and allowed to grow for a duration of eight days. Subsequently, the cells underwent three PBS washes, were fixed with methanol for 30 min, stained with crystal violet for another 30 min at ambient temperature, and then rinsed with distilled water to remove excess dye. Photographs of the plates were taken, and the number of colonies was quantitatively assessed and subjected to statistical analysis.

### 2.10 MTT Assay

Cells were plated at a density of 1 × 10^4^ cells per well on a 96‐well plate, with each condition replicated three times. The culture medium was refreshed every other day following a 24‐h period of drug treatment. The viability of the cells was quantified using the MTT assay. In brief, cells were treated with 50 μL of 0.2% MTT solution and incubated for 30 min at 37°C in a 5% CO_2_ environment. Absorbance was then measured at 560 nm using a plate reader (Dynex Technologies), and the data were subjected to analysis through a correlation test.

### 2.11 Flow Cytometry Analysis

After being incubated for 24 h, cells underwent a further 24‐h period of drug treatment. Post‐treatment, cells were collected, rinsed with PBS, and fixed in ice‐cold 70% ethanol for an overnight period. The fixed cells were then re‐suspended in PBS, which included 0.2 mg/mL of RNAse and 10 mM of PI, and left to incubate in the dark for 30 min at 37°Celsius. Analysis was conducted using a FACSCalibur flow cytometer (Becton Dickinson, CA), with the data subsequently subjected to a correlation test for evaluation.

### 2.12 Invasion Test

The cell invasion test employed matrigel invasion chambers (8 μm, 24‐well cell culture inserts) in alignment with the instructions provided by the manufacturer. A total of 5 × 10^4^ cells were suspended in 500 μL of medium and placed into the upper chamber, while 500 μL of medium supplemented with 10% FBS served as the chemoattractant in the lower chamber. Following a 22‐h period of drug treatment, cells present on the membrane's upper side were wiped off using a cotton swab, and the membranes underwent staining with crystal violet. The enumeration of cells was performed by calculating the average from cell counts across nine distinct 10× magnification fields.

### 2.13 RT‐qPCR and Western Blot

mRNA levels were quantified utilizing qPCR analysis, adhering to the guidelines provided by the manufacturer: Total RNA was extracted from cells using TRIzol reagent (Invitrogen, USA), following the manufacturer's instructions. After chloroform extraction and isopropanol precipitation, RNA pellets were washed with 75% ethanol, air‐dried, and dissolved in RNase‐free water. RNA purity and concentration were determined using a NanoDrop spectrophotometer (Thermo Fisher Scientific, USA). A total of 1 μg of RNA was reverse‐transcribed into complementary DNA (cDNA) using a PrimeScript RT Reagent Kit (Takara Bio, Japan) under the following conditions: 37°C for 15 min, followed by 85°C for 5 s. Quantitative PCR was performed using a SYBR Premix Ex Taq II (Takara Bio, Japan) in a 20 μL reaction volume containing 2 μL of cDNA, 10 μL of SYBR Green Mix, 0.4 μL of each primer (10 μM), and 7.2 μL of nuclease‐free water. The reaction was conducted on a QuantStudio 6 Flex Real‐Time PCR System (Applied Biosystems, USA). The thermal profile included an initial denaturation at 95°C for 30 s, followed by 40 cycles of 95°C for 5 s and 60°C for 30 s. Melting curve analysis was performed to verify amplification specificity. Relative mRNA levels were calculated using the 2^(‐ΔΔCt) method, normalized to the expression of GAPDH as an internal control.

Similarly, Western blot analysis was conducted in accordance with the manufacturer's instructions: Cells were harvested and lysed in RIPA lysis buffer (Beyotime, China) supplemented with phenylmethylsulfonyl fluoride (PMSF, 1 mM), phosphatase inhibitors, and protease inhibitor cocktail. The lysates were incubated on ice for 30 min and then centrifuged at 12000 × g for 15 min at 4°C. The supernatant was collected, and protein concentration was determined using a BCA Protein Assay Kit (Beyotime, China). Equal amounts of protein (30 μg) were separated by SDS‐PAGE on 10%–12% polyacrylamide gels. The separated proteins were transferred to PVDF membranes (Millipore, USA) using a semi‐dry transfer system (Bio‐Rad, USA). Membranes were blocked with 5% non‐fat milk in TBST (TBS with 0.1% Tween‐20) for 1 h at room temperature. Primary antibodies against the target protein and loading control (AR and GAPDH) were diluted in blocking solution and incubated overnight at 4°C. After washing, membranes were incubated with HRP‐conjugated secondary antibodies for 1 h at room temperature. Protein bands were visualized using an enhanced chemiluminescence (ECL) detection kit (Tanon, China) and imaged with a Tanon 5200 chemiluminescence imaging system (Tanon, China). Band intensities were measured using ImageJ software (NIH, USA). The relative protein expression levels were calculated as the ratio of the target protein to the loading control. The relative mRNA and protein expression levels were analyzed for correlation using Pearson correlation analysis. Data were presented as mean ± standard deviation (SD) and analyzed using GraphPad Prism 9 software. A *p* value < 0.05 was considered statistically significant.

### 2.14 The Kaplan–Meier Plotter Analysis

The Kaplan–Meier Plotter is a versatile tool that evaluates the correlation between the expression of various biomolecules (mRNA, miRNA, protein, and DNA) and survival outcomes across more than 35 000 samples from 21 different tumor types. We employed this tool to perform clinical survival prognosis analysis on our screening results, further exploring the correlations between specific parameters through stratified analysis [45].

### 2.15 Construction of Survival Prediction Model

We downloaded STAR‐counts data and corresponding clinical information for hepatocellular carcinoma (HCC) patients from the Cancer Genome Atlas (TCGA) database. Next, we extracted data in the TPM (transcripts per million) format and performed log2(TPM + 1) normalization. After retaining samples with both RNA sequencing data and clinical information, a total of 371 samples were included for subsequent analysis. First, univariate and multivariate Cox proportional hazards regression analyses were conducted, and a forest plot was generated using the “forestplot” R package to visually present the *p* values, hazard ratios (HRs), and 95% confidence intervals (CIs) for each variable. Subsequently, based on the results of the multivariate Cox proportional hazards model, a nomogram was constructed using the “rms” R package to predict overall recurrence rates at 1–3 years. Statistical analyses were performed using R software (version 4.0.3), and results were considered statistically significant when the *p* value was less than 0.05 [46].

### 2.16 Ethical Statement

The study was conducted in accordance with the Declaration of Helsinki. Bioinformatic analysis was obtained from the Ethics Committee of the First Affiliated Hospital of Medical School of Ningbo University.

### 2.17 Statistical Analysis

Statistical outcomes are depicted as the mean ± SD from at least three independent biological experiments. Differences between the two groups were determined using Student's *t*test, and differences among more than two groups were assessed via one‐way analysis of variance (ANOVA). The Kaplan–Meier method was utilized for analyzing overall survival curves, which were then compared using the log‐rank test. To determine the relationships between two variables, Spearman's rank correlation coefficient was employed. These analyses were performed using SPSS software (V20, IBM Corp., Armonk, NY, USA) and GraphPad Prism version 8.0. The figures indicate statistical significance at *p* < 0.05 (*), *p* < 0.01 (**), *p* < 0.001 (***), and *p* < 0.0001 (****).

## 3 Results

### 3.1 Identifying Active Metformin Compounds and Ferroptosis‐Related Target Genes in Liver Cancer

**FIGURE 2:**
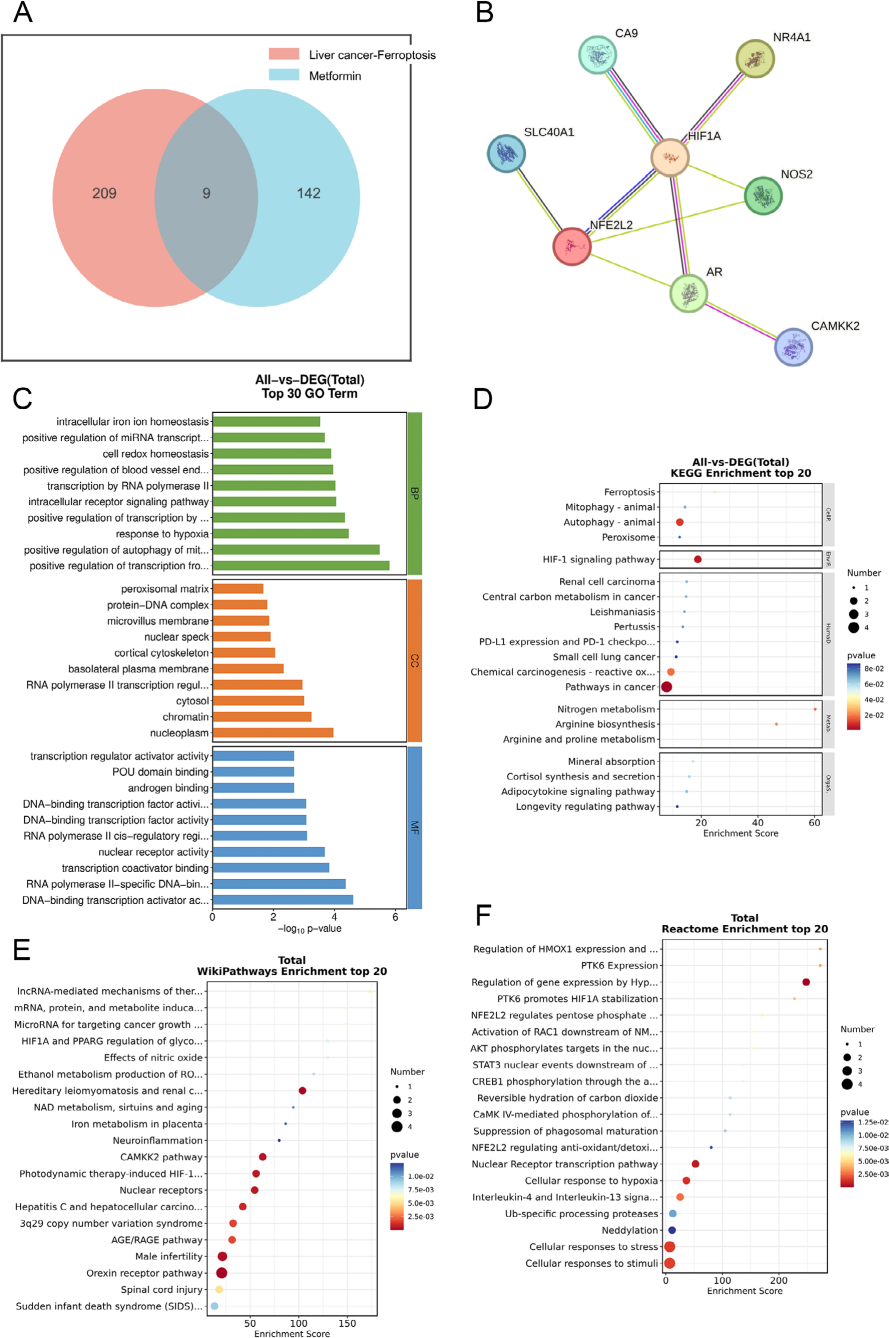
Identification targets of Metformin–Liver cancer. (A) Intersection results of Metformin drug target genes and liver cancer ferroptosis‐related target genes. (B) PPI network. The circle nodes represent core targets, and the connecting lines represent varying degrees of correlation. (C) GO enrichment analysis. (D) KEGG enrichment analysis. (E) Wiki enrichment analysis. (F) Reactome enrichment analysis.

We first obtained the 3D structure of Metformin from PubChem and submitted it to the Swiss Target Prediction, SuperPred, and Similarity Ensemble Approach databases. After deduplication, we obtained 151 drug target genes of Metformin. At the same time, we used the GeneCards database to obtain 18 753 liver cancer target genes. We also selected 348 ferroptosis suppressor target genes from the ferroptosis database. Using the OEBiotech Cloud tool to intersect the two sets, we obtained a total of 218 liver cancer ferroptosis target genes. A further cross‐analysis of the potential targets of Metformin and liver cancer ferroptosis identified nine intersecting targets as candidate targets for the treatment of liver cancer ferroptosis by Metformin (Figure 2A).

### 3.2 PPI Analysis

The construction of a protein–protein interaction network (PPI) was advanced using STRING, where disconnected nodes were eliminated, yielding a network of 8 nodes and 48 edges. The targets were prioritized by betweenness and centrality metrics to pinpoint hub proteins. The refined pivotal targets comprised NFE2L2, AR, HIF1A, CA9, and NR4A1. These key genes were subsequently utilized in molecular docking investigations (Figure 2B).

### 3.3 GO Function, KEGG Pathway, Wiki Pathway, and Reactome Enrichment Analysis

Following the exclusion of inactive component targets and disease targets, the Bioconductor package within R software was employed to conduct GO analysis on the collected data. This analysis identified significantly enriched biological processes, which were then illustrated in the figure. The scope of GO enrichment encompasses biological processes, cellular components, and molecular functions. It was discovered that the target genes in question are predominantly associated with the positive regulation of transcription from the RNA polymerase II promoter in response to hypoxia, the cortical cytoskeleton, and transcription regulator activator activity (Figure 2C). Moreover, enrichment analyses via KEGG and Wiki pathways revealed that these genes are chiefly concentrated in pathways including nitrogen metabolism, the arginine biosynthesis signaling pathway, and mechanisms of therapeutic resistance mediated by lncRNA, as well as the induction pathways for mRNA, protein, and metabolites by cyclosporin A signaling pathways (Figure 2D,E). Remarkably, Reactome enrichment analysis suggested that genes pertaining to PTK6 expression and the regulation of HMOX1 expression and activity could represent one of the mechanisms through which metformin acts in the treatment of liver cancer (Figure 2F).

### 3.4 DCTD Network Analysis

**FIGURE 3:**
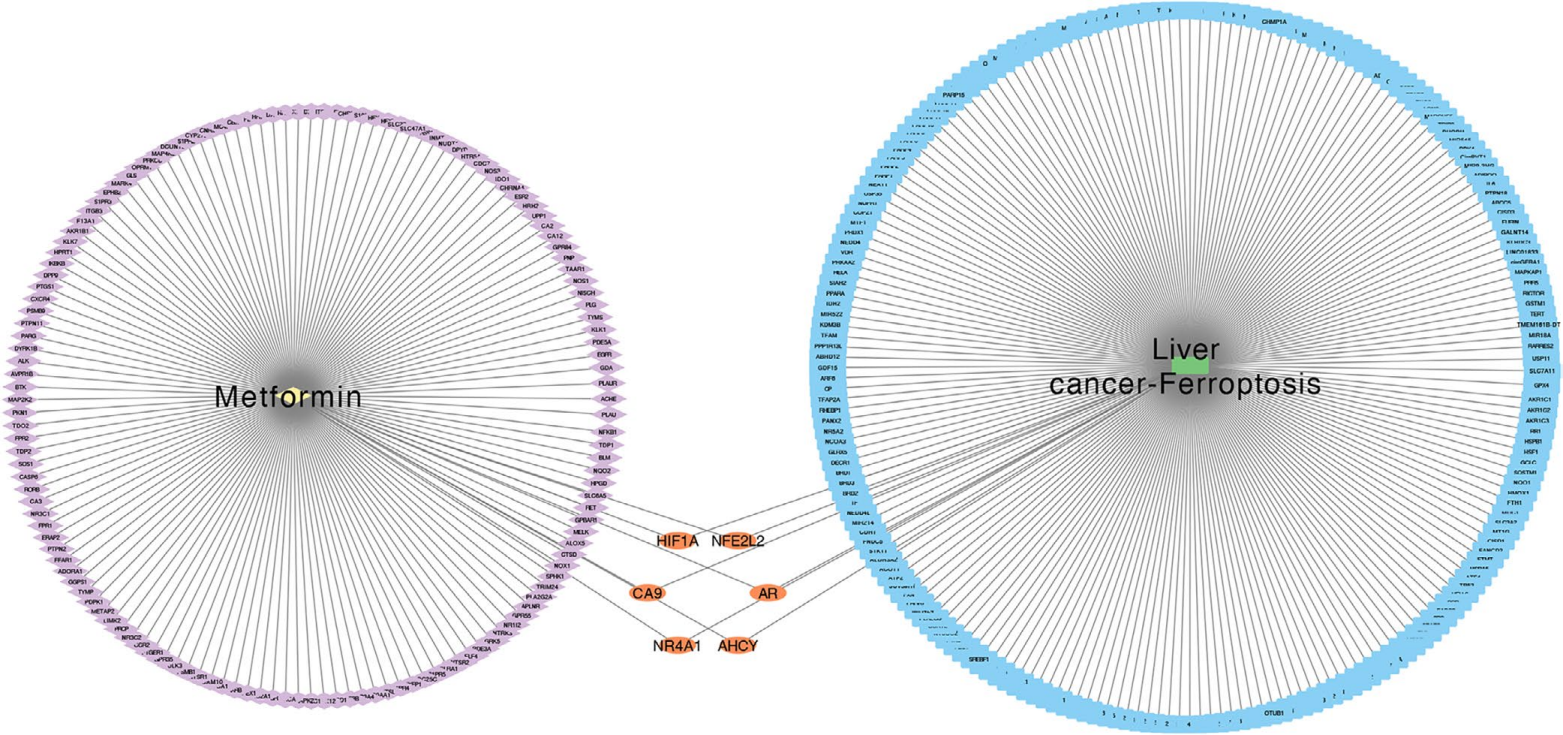
DCTD network and enrichment analysis. Topological network connection results of drugs, active compounds, intersecting genes, and diseases.

Building on the findings, a DCTPD (Drug–Compound–Target–Pathway–Disease) network was developed using Cytoscape version 3.9.0. The figure presents the intricate interactions involved in Metformin's role in promoting ferroptosis within liver cancer. Nodes, distinguished by various colors and shapes, denote diseases, active components, targets, pathways, and drugs, with the quantity of connections to a node signifying its significance within the network. The analysis reveals a network where Metformin is linked to six potential targets, showcasing how a single active component may interact with multiple targets. This network adeptly illustrates the comprehensive multi‐component and multi‐target intervention capacity of Metformin in triggering ferroptosis in liver cancer (Figure 3).

### 3.5 Analysis of Molecular Docking and Molecular Dynamics Simulation Results

**FIGURE 4:**
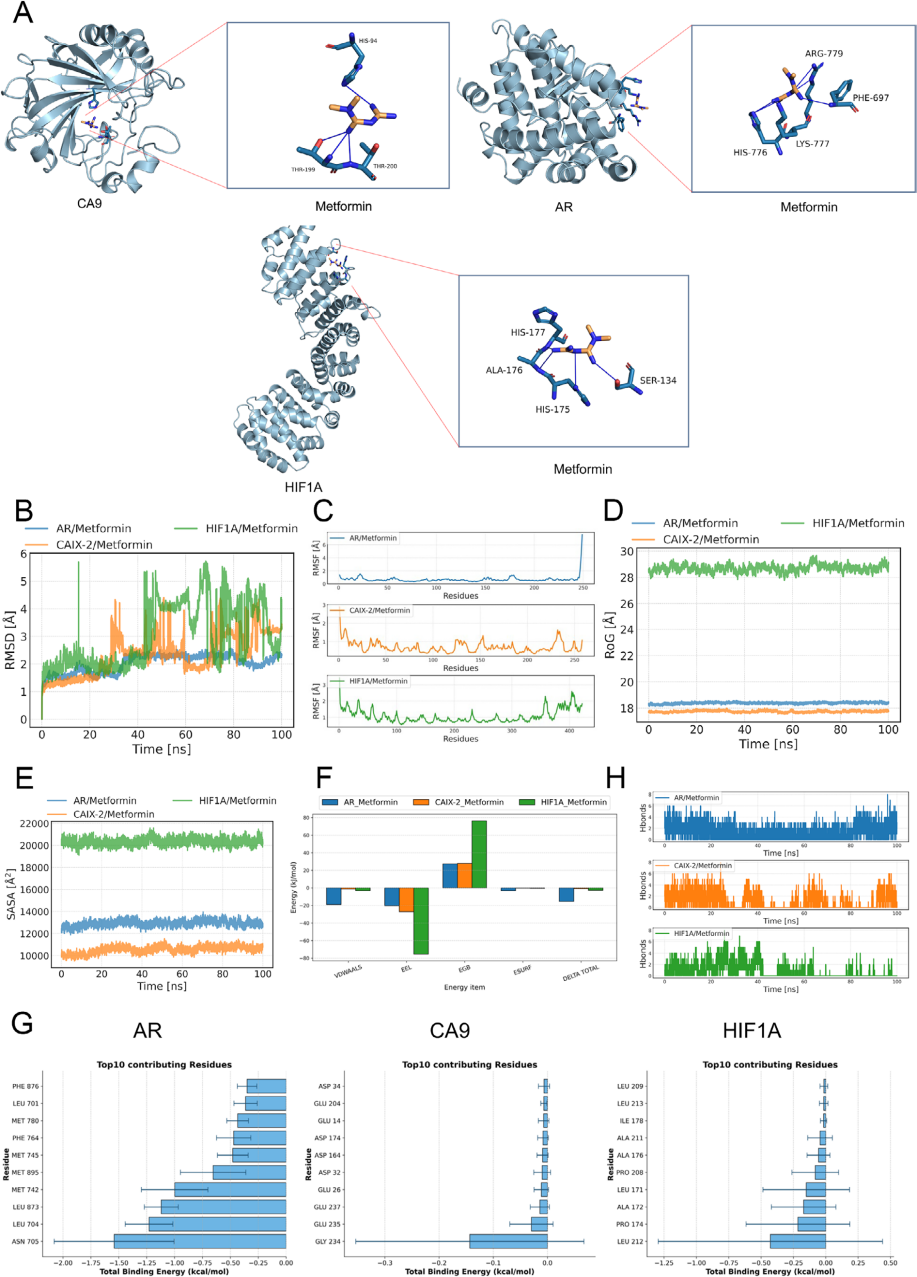
3D docking patterns and molecular dynamics simulation results. (A) Metformin binding to CA9, AR and HIF1A crystal structure. (B) The variation of root mean square deviation (RMSD) of the complex over time during molecular dynamics simulation. (C) Root mean square fluctuation (RMSF) calculated based on molecular dynamics simulation trajectories. (D) The variation of the radius of gyration (RoG) calculated based on molecular dynamics simulation trajectories over simulation time. (E) The solvent‐accessible surface area (SASA) of each complex during the molecular dynamic simulation. (F) Binding energy results. (G) Top 10 contributing amino acids in the molecular protein complex. (H) The variation in the number of hydrogen bonds between the small molecule and the protein during the molecular dynamics simulation.

To delve deeper into the potential therapeutic effects of Metformin and to validate network pharmacology outcomes, we proceeded with molecular docking studies on 6 selected targets, AR, HIF1A, NFE2L2, NR4A1, AHCY, and CA9(CAIX‐2), based on prior enrichment analysis findings. Using AutoDock for calculating docking affinity scores and ligand efficiency, we considered scores below −4.25 kcal/mol as reflective of favorable docking affinity, and a higher ligand efficiency is considered more ideal because it indicates that each atom contributes more binding free energy, thereby improving the molecule's binding efficiency. The better scores of 3 proteins among the above 5 targets are outlined in Table 1. We also created and illustrated 3D diagrams of their molecular interactions (Figure 4A).

**TABLE 1:** Target molecule docking results for candidate active components.

Metformin			
Compound name	AR	HIF1A	CA9
Affinity (kcal/mol)	−4.9	−5.0	−4.5
Ligand Efficiency	−0.54	−0.56	−0.53

The RMSD of molecular dynamics (MD) simulations reflects the motion of the complex; larger and more fluctuating RMSD values indicate more intense motion, whereas smaller and steadier RMSD values suggest more stable motion. As shown in Figure 4B, the RMSD profiles of the AR‐Metformin, CA9‐Metformin, and HIF1A‐Metformin systems during the simulation process were analyzed. The AR‐Metformin system exhibited good convergence and stable fluctuations throughout the simulation, indicating stable binding. In contrast, the CA9‐Metformin and HIF1A‐Metformin systems showed larger fluctuations and poorer dynamic convergence, suggesting weaker binding stability. RMSF reflects the flexibility of the protein during the MD simulation. Typically, upon drug binding, the protein's flexibility decreases, contributing to its stabilization and enhancement of enzymatic activity. As shown in Figure 4C, RMSF values of all proteins (except at the termini) decreased after Metformin binding, indicating that the core structure of the proteins remained rigid. In particular, the AR protein exhibited the lowest RMSF, suggesting reduced flexibility upon ligand binding. Overall, low protein flexibility provides a foundation for stable protein‐ligand binding. The radius of gyration (RoG) comparison shows that the AR‐Metformin system had a RoG value of approximately 18 Å with minimal fluctuations, indicating a compact and stable structure. In contrast, the CA9‐Metformin system appeared more compact (approximately 17.5 Å), but had weaker binding energy, indicating potentially lower stability. The HIF1A‐Metformin system displayed a significantly larger RoG value (approximately 28 Å), suggesting a more open binding site and reduced stability. Overall, the AR system demonstrated better binding compactness and stability, making it a potentially superior binding target (Figure 4D). The comparison of solvent‐accessible surface area (SASA) revealed that the HIF1A‐Metformin system had the highest SASA value (~22 000 Å^2^), indicating a more open structure with a larger solvent‐exposed area. The AR‐Metformin system showed a moderate SASA value (~14 000 Å^2^) with minimal fluctuations, reflecting a stable and moderately solvent‐exposed structure. The CA9‐Metformin system had the lowest SASA value (~10 000 Å^2^), indicating the most compact structure. In conclusion, the AR system displayed good stability and moderate solvent exposure, which might favor ligand binding and biological activity (Figure 4E).

Based on the molecular dynamics (MD) simulation trajectories, binding energies were calculated using the MM‐GBSA method, which provides a more accurate reflection of the binding mode between small molecules and target proteins. As shown in Table 2, the binding energies of the AR‐Metformin, CA9‐Metformin, and HIF1A‐Metformin complexes were − 15.39 ± 1.20, −0.87 ± 1.53, and − 2.78 ± 4.26 kcal/mol, respectively. Negative values indicate binding affinity between the molecules and target proteins, with lower values representing stronger binding. Clearly, the results demonstrate that AR‐Metformin exhibits strong binding affinity. The binding energy of AR‐Metformin was primarily contributed by van der Waals and electrostatic energies, while the solvation energy was unfavorable for binding. In contrast, CA9‐Metformin and HIF1A‐Metformin showed weaker binding energies.

**TABLE 2:** Binding free energies and energy components predicted by MM/GBSA (kcal/mol).

System	ΔE_vdW_	ΔE_elec_	ΔG_GB_	ΔG_SA_	ΔG_bind_
AR_Metformin	−19.06 ± 1.42	−20.49 ± 7.54	27.35 ± 7.36	−3.19 ± 0.10	−15.39 ± 1.20
CAIX‐2_Metformin	−1.37 ± 1.95	−27.10 ± 37.40	27.88 ± 38.15	−0.28 ± 0.41	−0.87 ± 1.53
HIF1A_Metformin	−3.14 ± 4.79	−75.57 ± 34.13	76.40 ± 35.25	−0.48 ± 0.71	−2.78 ± 4.26

*Note:* Δ*E*_elec_: electrostatic energy; Δ*E*_vdW_: van der Waals energy; Δ*G*_GB_: electrostatic contribution to solvation; Δ*G*_SA_: non‐polar contribution to solvation; Δ*G*_bind_: binding free energy.

In the AR‐Metformin, CA92‐Metformin, and HIF1A‐Metformin systems, the energy contributions of amino acid residues revealed different binding characteristics. The binding energy of the AR system was predominantly driven by ASN‐705, along with contributions from LEU 873, LEU 701, and MET 742, indicating that both electrostatic and hydrophobic interactions are key driving forces for ligand stability. In the CA9 system, negatively charged residues (e.g., GLU 237, ASP 32, and GLU 26) contributed significantly to the energy, suggesting that electrostatic interactions and hydrogen bonds are the primary binding mechanisms. Comparatively, the binding site of the HIF1A system was dominated by hydrophobic residues (e.g., LEU 212, PRO 174, and ALA 172), showing hydrophobic binding characteristics similar to the AR system. Based on the absolute binding energy values, AR and HIF1A exhibited stronger binding trends, while the binding energy contribution of CA9 was weaker, suggesting that Metformin may have higher binding affinity and stability in the AR and HIF1A systems (Figure 4F,G).

As shown in Figure 4H, the dynamic characteristics of hydrogen bonds in the AR‐Metformin, CA92‐Metformin, and HIF1A‐Metformin systems differed significantly. The AR‐Metformin system maintained a relatively stable number of hydrogen bonds throughout the simulation, with an average of 2–4 hydrogen bonds, indicating sustained and stable hydrogen bonding interactions between the ligand and protein. In the CA9‐Metformin system, hydrogen bond fluctuations were more pronounced, with increased numbers observed during the 20–40 ns and 60–80 ns time frames, although the overall number remained low, indicating dynamic formation and breaking of hydrogen bonds during binding. The HIF1A‐Metformin system showed significant fluctuations in the number of hydrogen bonds, with frequent peaks (approaching 6–8 bonds), suggesting the presence of strong but short‐lived hydrogen bonding interactions, albeit with slightly lower overall stability compared to AR‐Metformin. These differences highlight the distinct characteristics of hydrogen bonding interactions across the protein‐ligand systems. Such results suggest Metformin's potential in targeting ferroptosis suppressor proteins within liver cancer, although more empirical studies are required for confirmation.

### 3.6 In Vitro Validation Experiments on the Inhibition of Liver Cancer Cells by Metformin

**FIGURE 5:**
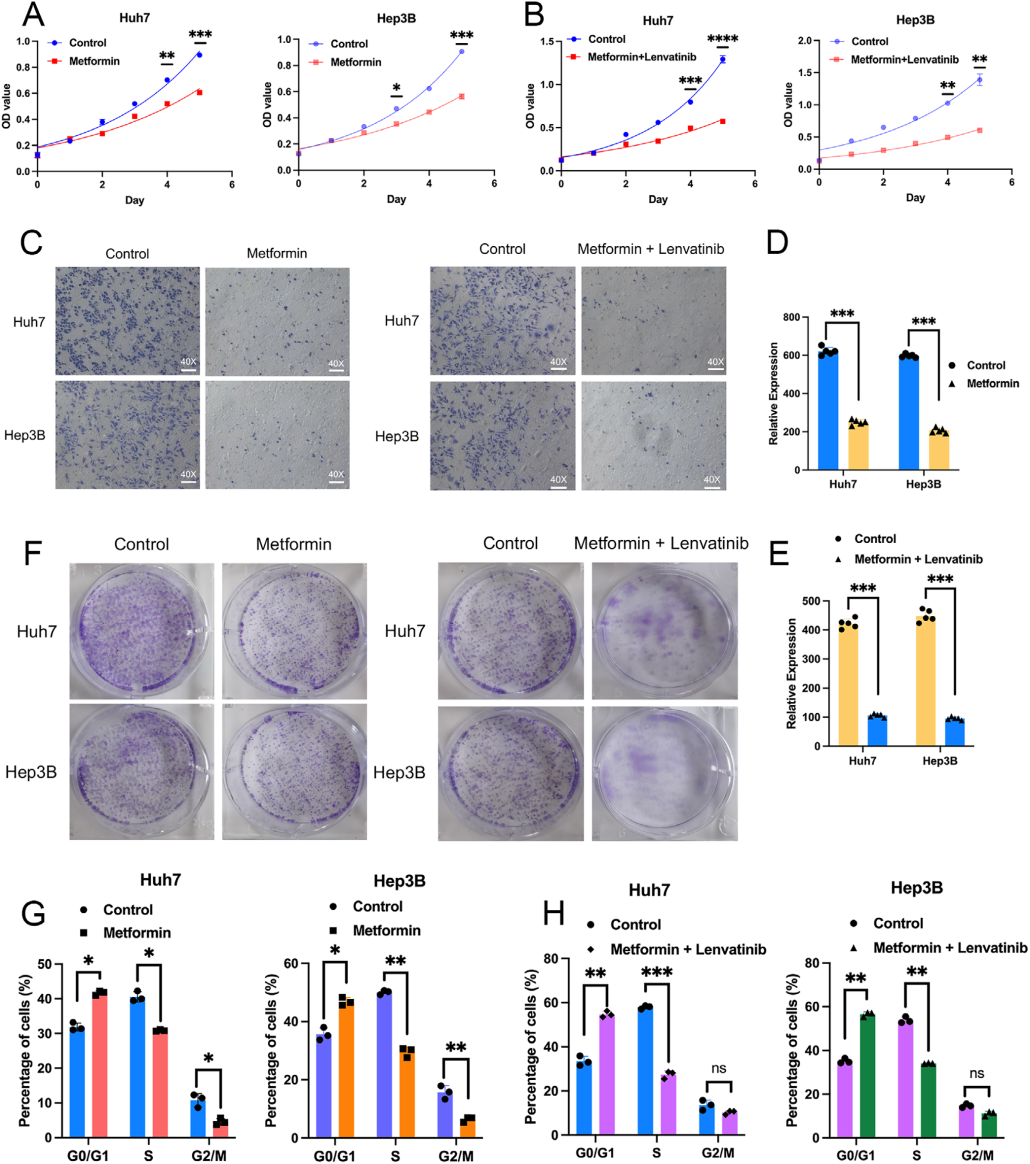
In vitro validation experiments of Metformin alone or in combination with Lenvatinib inhibiting liver cancer cells. (A, B) Proliferation rates of Control, Metformin alone, and Metformin combined with Lenvatinib, in Huh7 and Hep3B cells treated as indicated were measured by MTT assay. (C, D, E) Matrigel invasion chambers were used to measure the invasiveness of each group of cells treated as indicated, then stained with crystal violet and photographed (Magnification, ×200 for cells; scale bar, 100 μm). (F) After 10 days, cells were stained with crystal violet and imaged to assess their colony formation ability. (G, H) The cell cycle distribution of each group of cells was analyzed by flow cytometry and treated as indicated.

Building upon the preliminary confirmation that Metformin targets the protein CA9, we further conducted in vitro validation experiments using liver cancer cells to evaluate the therapeutic effects of Metformin alone and in combination with Lenvatinib on liver cancer. We assessed the inhibitory effects of Metformin on liver cancer cells through growth curves, clonogenic assays, invasion experiments, and cell cycle analysis. The results showed that after 24 h of Metformin treatment, the growth rate of liver cancer cells Huh7 and Hep3B was significantly slowed down after three or four days, respectively (Figure 5A,B). Additionally, the inhibitory effect of Metformin in combination with Lenvatinib became even more pronounced after 3 days. Results from clonogenic and invasion assays also indicated that both Metformin alone and in combination with Lenvatinib could significantly inhibit the clonogenic and invasive capabilities of Huh7 and Hep3B cells (Figure 5C–F). Finally, cell cycle analysis revealed that after Metformin treatment, liver cancer cells were primarily arrested in the G0/G1 phase, while the S phase and G2/M phase were relatively reduced compared to the control group (Figures 5G, S1, and S2). The inhibitory effect on liver cancer cells was further enhanced after combination with Lenvatinib (Figure 5H). These findings suggest that Metformin alone and in combination with Lenvatinib can inhibit the growth rate, clonogenic ability, invasiveness, and slow down the cell cycle progression of the in vitro liver cancer cells Huh7 and Hep3B. Therefore, Metformin can slow down disease progression by inhibiting liver cancer cells.

### 3.7 Metformin Demonstrates Anticancer Activity by Suppressing Ferroptosis Suppressor Protein Expression in Liver Cancer Cells

**FIGURE 6:**
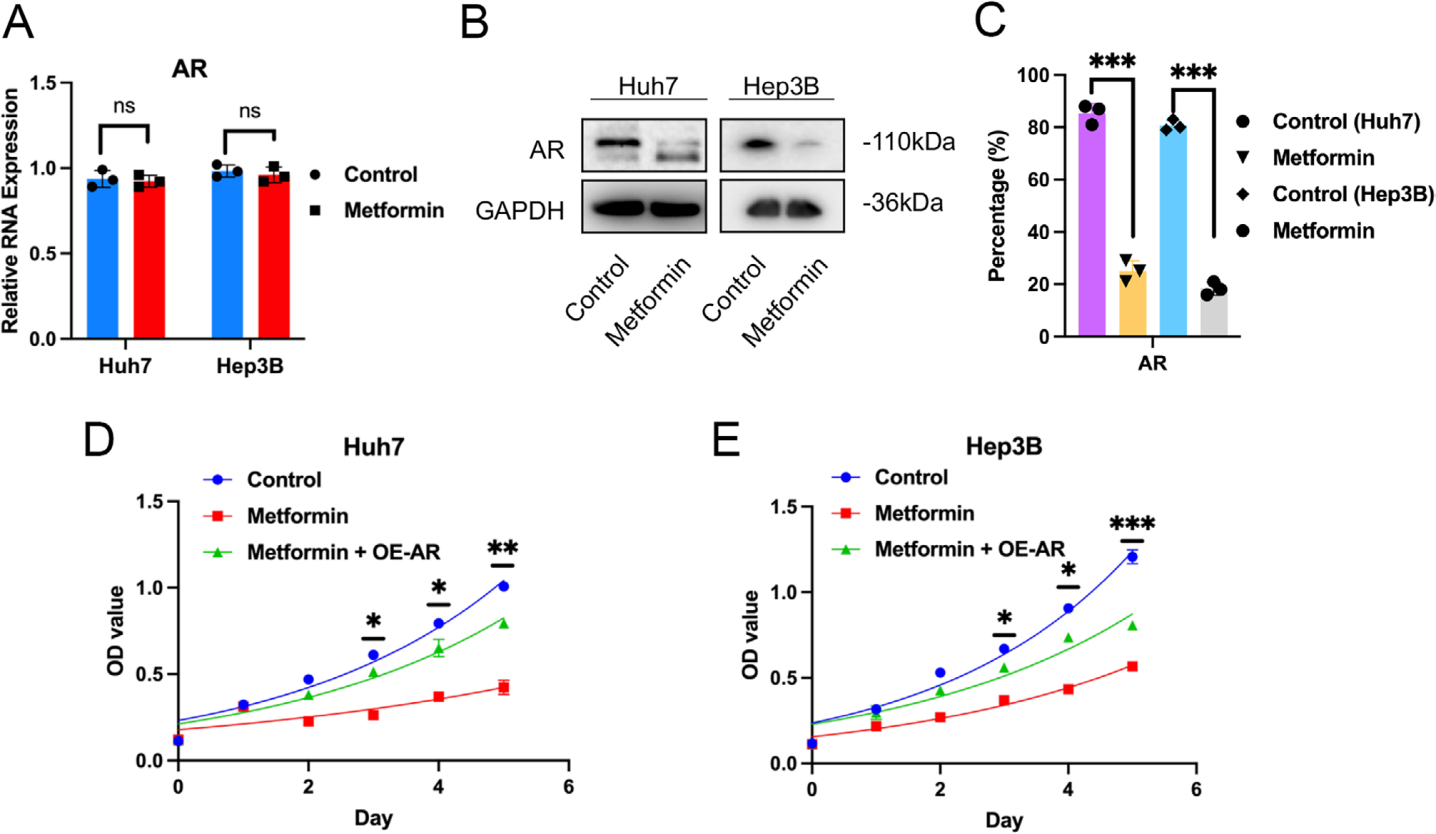
AR is highly expressed in liver cancer tissues, and its protein expression level can be downregulated by Metformin. (A, B, C) The PCR and western blot results of AR in Huh7 and Hep3B cells were treated as indicated. (D, E) Proliferation rates of Control, Metformin alone, and Metformin combined with overexpression of AR, in Huh7 and Hep3B cells treated as indicated were measured by MTT assay.

Following the confirmation from cellular functional assays, we delved deeper into Metformin's suppressive action on the docking protein AR, employing PCR, Western blot, and cell function analyses. Results from in vitro cellular experiments illustrated that Metformin notably reduced the protein expression levels of the ferroptosis suppressor protein AR, although its impact on mRNA levels was not significant (Figure 6A–C). Secondly, the cell growth curve results showed that after metformin treatment, the growth rate of the two hepatocellular carcinoma cell lines was significantly reduced compared to the control group. However, the inhibitory effect of metformin was partially attenuated after overexpression of the AR gene (Figure 6D,E). These findings highlight Metformin's capability to thwart the progression and emergence of liver cancer through the induction of ferroptosis.

### 3.8 High Expression of AR in Liver Cancer Patients Is Associated With Better Prognosis

**FIGURE 7:**
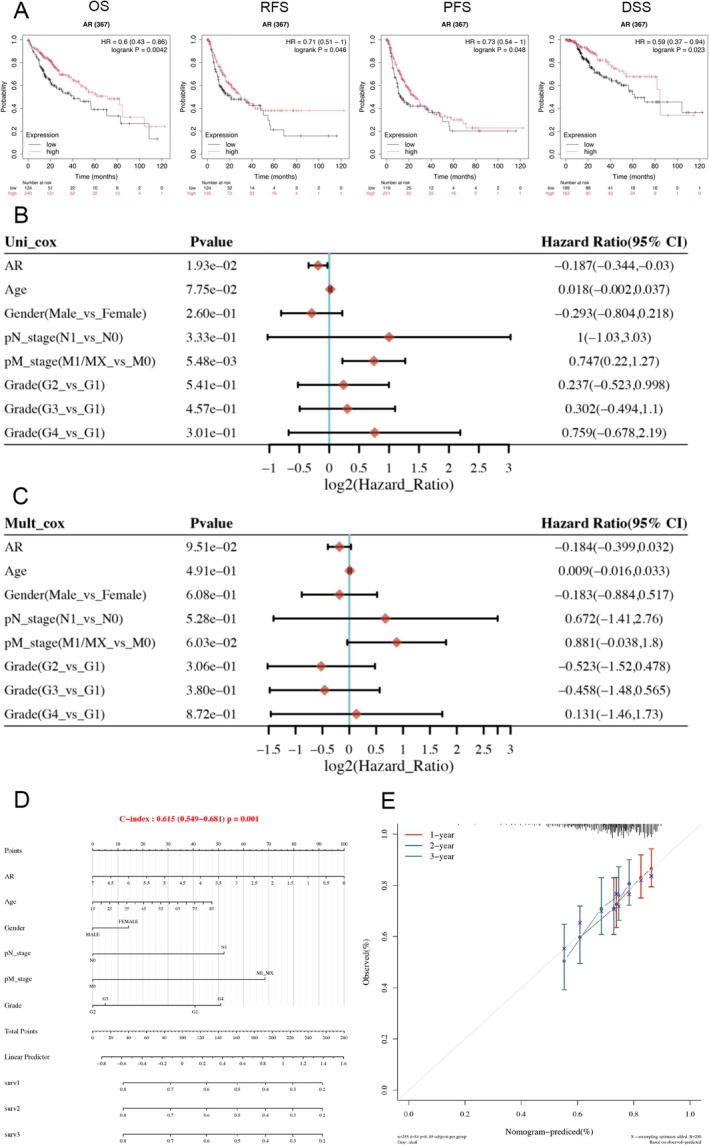
Clinical prognostic potential of four ferroptosis suppressor genes in liver cancer patients. (A) Kaplan–Meier survival analysis of AR for four outcomes in HCC patients. (B, C) Univariate and multivariate Cox analyses provide the *p* values, hazard ratios (HR), and confidence intervals (CI) for AR expression and different clinical characteristics. (D) The nomogram can predict the 1‐year, 2‐year, and 3‐year overall survival of HCC patients. (E) The calibration curve of the overall survival nomogram model in the discovery cohort is shown, where the diagonal dashed line represents the ideal nomogram, and the blue, red, and orange lines represent the observed 1‐year, 2‐year, and 3‐year nomograms, respectively.

In the clinical application aspect, we selected AR to construct a liver cancer ferroptosis prediction model. Firstly, we downloaded gene and clinical data of hepatocellular carcinoma (HCC) patients from the K‐M database. Based on AR expression levels, the patients were divided into high and low expression groups, and K‐M curve analyses were performed for four outcomes: OS, RFS, PFS, and DSS. The results showed that patients in the high AR expression group had better prognoses in all four outcomes compared to the low expression group, with statistically significant differences (*p* < 0.05, Figure 7A).

Subsequently, we conducted univariate and multivariate Cox regression analyses of clinical indicators and AR expression with OS. The results revealed that AR expression, age, gender, pathological N stage, pathological M stage, and grade were independent prognostic factors for HCC patients (Figure 7B,C). Based on these factors, we constructed a nomogram to predict the 1‐ to 3‐year survival probability of these HCC patients (Figure 7D). The nomogram showed a C‐index of 0.615, with statistical significance (*p* = 0.001).

Finally, we developed calibration curves for the nomogram, and the results for 1‐ to 3‐year predictions closely aligned with the ideal values (Figure 7E).

## 4 Discussion

Ferroptosis is a new type of non‐apoptotic and non‐necrotizing process of cell death, which is characterized by irondependence. Its morphological features mainly include complete fine nuclei, non‐aggregation of chromophores, non‐rupture and vesiculation of the plasma membrane, reduction or disappearance of mitochondria, rupture of the outer membrane, and increased density of the inner membrane et al. [45]. In addition to the above characteristics, the signaling pathway in tumor cells is also different from the path of apoptosis and other death processes [46]. The controlling genes of ferroptosis also differ from those controlling other cell death modes, mainly including ribosomal albumin L8, iron response element binding protein two, citrate synthetase (CS), tetrapeptide repeat domain 35, F0 complex subunit C3, and human acyl‐CoA synthetase 2 mitochondrial 6 protein‐coding genes, which regulate iron uptake, iron metabolism, and iron storage by regulating iron ion levels [47]. Besides, the biochemical process of ferroptosis is marked by increased concentrations of lipid peroxide and ferrous ions, which can be explained by the abnormal metabolism of lipid oxidants in cancer cells under the catalysis of iron ions and the destruction of the original redox balance, then attacking biological macromolecules and leading to cell death. The progress is specifically manifested in the inactivation of glutathione peroxidase 4 (GPX4) and the deposition of lipid peroxides [48]. In addition, cysteine availability, glutathione (GSH) biosynthesis, polyunsaturated fatty acid metabolism, and phospholipid regulation are the key factors in the ferroptosis process [45].

The liver is an important organ for storing and metabolizing iron, and iron balance plays an important role in maintaining the normal physiological function of the human body. Excessive deposition of iron in the liver can cause liver damage and further lead to hepatic lobular fibrosis, cirrhosis, and liver cancer. Iron can also promote the damage of alcohol to the liver, which is related to the oxygenation reaction, enhancing the lipid peroxidation reaction, and stimulating lipocytes [49, 50]. GABUTTI et al. have reported that long‐term use of chelate could reverse complications associated with iron overload, such as liver fibrosis [51]. Liver fibrosis is a secondary reaction after irreversible dysfunction of the cytoplasm, nucleus, and other organelles [52, 53, 54]. Besides, ferroptosis is considered a strong tumor growth inhibitor. In colon cancer, lung cancer, central nervous system malignancy, melanoma, oocyte carcinoma, and breast adenocarcinoma, the effect of ferroptosis enhances the sensitivity of tumors to chemotherapy drugs [55]. However, the specific function of iron death in HCC has not been fully explained, and previous research has found that the cytotoxic effects of Sorafenib (a multi‐kinase inhibitor) in HCC could be recognized by the start of oxidative stress and ferric death, indicating that iron death plays an important role in the mechanism of drug‐induced death of HCC cells [56, 57, 58, 59].

At present, the exploration of Metformin's molecular mechanisms in treating liver cancer is largely focused on cell‐based studies, supplemented by some animal research. The primary therapeutic strategies of Metformin involve the suppression of liver cancer stem cell proliferation, activation of the AMPK signaling pathway, engagement of tumor suppressor genes while repressing oncogenes, induction of liver cancer cell apoptosis, and alterations in epigenetic factors, among others [60, 61]. In the realm of ferroptosis research, publications on Metformin's effects on liver cancer are comparatively sparse. For instance, Zhang et al. demonstrated that Metformin could suppress PPARGC1A expression in liver cancer cells through an m6A‐dependent mechanism [62]. Furthermore, Hu and associates showed that Metformin could enhance the sensitivity of liver cancer cells to Sorafenib via the ATF4/STAT3 signaling pathway [63]. Our research suggests that Metformin may curb the proliferation and spread of liver cancer cells by targeting the ferroptosis suppressor protein AR.

In recent years, with the deepening understanding of tumor biology and targeted therapies, the androgen receptor (AR) has attracted considerable attention. While substantial research on AR in prostate cancer continues to evolve, studies have expanded beyond prostate cancer to explore AR's role in other tumors, such as melanoma, gastric cancer, hepatocellular carcinoma (HCC), and breast cancer [64, 65]. Interestingly, AR can exhibit both tumor‐promoting and tumor‐suppressing effects depending on the context. AR's role varies across different tumor types and even among subtypes of the same tumor. Additionally, AR can indirectly promote tumor progression by inhibiting the activity and stemness of infiltrating CD8+ T cells. AR is implicated in the pathogenesis of hepatocellular carcinoma (HCC). Approximately 37% of HCC tumors exhibit nuclear overexpression of AR, which is significantly associated with advanced disease stages and poor survival outcomes. Overexpression of AR in HCC cells significantly alters the AR‐dependent transcriptome, thereby promoting the growth of oncogenic cells [66]. In VETC+ (vascular encapsulated tumor cluster‐positive) HCC cells, AR overexpression inhibits VETC formation and intrahepatic metastasis. AR appears to suppress VETC‐dependent intrahepatic metastasis by downregulating angiopoietin‐2 (Angpt‐2) expression and VETC formation. On the other hand, AR may promote lung metastasis in VETC+ HCC and both hepatic and pulmonary metastases in VETC− HCC by upregulating Rac1 expression [67]. This study found that the AR protein could bind to the Metformin molecule with a high binding score. Additionally, in liver cancer cells treated with Metformin, the protein expression level of AR was downregulated, suggesting that the AR protein might be a new target of Metformin. More in vivo and in vitro mechanistic studies may be needed for validation in the future.

## 5 Conclusions

In this research, we identified molecular targets for Metformin‐induced ferroptosis in liver cancer through network pharmacology docking screening, followed by validation with in vitro cell experiments. Subsequently, we constructed a liver cancer ferroptosis‐related prediction model using a combination of four ferroptosis suppressor genes. Our findings provide new subsequent targets for Metformin‐induced ferroptosis in liver cancer, but further in‐depth validation and exploration of the specific mechanisms of action are required.

## Author Contributions

Siming Zheng designed the research. Bin Zhang and Zehao Yu carried out the experiments. Jinghui Zhang and Yini Xu undertook data analyses. Mengna Zhang and Ziqi Dai wrote the manuscript. Jiyun Zhu reviewed the manuscript and supervised the study. All authors approved the final version of the manuscript for submission.

## Conflicts of Interest

The authors declare no conflicts of interest.

## Supporting information


Data S1.

## Data Availability

The datasets generated and/or analyzed during the current study are not publicly available as they are maintained in a HIPAA compliant database at the study institution. HIPAA compliant data can be made available from the corresponding author on reasonable request.
