# Supplementary material for: Identification of Androgen Receptor as a Molecular Docking Target for Survival and Response to Metformin‐Induced Ferroptosis in Liver Cancer
Source: Cancer Rep (Hoboken). 2025 Jun 30;8(7):e70245. doi: 10.1002/cnr2.70245 (PMC12207094; doi:10.1002/cnr2.70245)
Supplement: Supplementary file 1 — Data S1. [file CNR2-8-e70245-s001.docx]

**Supplemental Material**

**S-Figure 1.** Each image represents the results of cell cycle experiments on HCC cells Huh7 and Hep3B under different treatment conditions. The various peaks in the figure represent the different cell cycle outcomes of the two liver cancer cell lines following metformin treatment, with the right side showing the proportion of each phase in the entire cell cycle.

**S-Figure 2.** Each image represents the results of cell cycle experiments on HCC cells Huh7 and Hep3B under different treatment conditions. The various peaks in the figure represent the different cell cycle outcomes of the two liver cancer cell lines following Metformin and Lenvatinib treatment, with the right side showing the proportion of each phase in the entire cell cycle.
